# Dataset on the cyclic experimental behavior of Steel frames with Reinforced Concrete infill Walls

**DOI:** 10.1016/j.dib.2018.06.111

**Published:** 2018-06-30

**Authors:** Francesco Morelli, Silvia Caprili, Walter Salvatore

**Affiliations:** Department of Civil and Industrial Engineering – University of Pisa, Italy

## Abstract

This paper presents the experimental data on the cyclic behavior of Steel frames with Reinforced Concrete infill Walls (SRCW). Two specimens, characterized by a different shear studs distribution, have been tested: the first one is provided with shear studs positioned only in the four corners of the steel frame; the second one presents shear studs all distributed along the perimeter of the steel frame except for the zone of the dissipative fuses. The overall setup, loading protocol, collapse mechanisms, force-displacement curves for both the whole system and the main single components are described for the two tested prototypes.

**Specifications Table**

**Table:** 

Subject area	*Engineering*
More specific subject area	*Earthquake Engineering*
Type of data	*Table, figures*
How data was acquired	*Displacements were acquired through potentiometric displacement sensors, deformations through linear strain gauges, forces through load cells.*
Data format	*Analyzed*
Experimental factors	*The concrete was cast two months before the test*
Experimental features	*Cyclic global force-displacement curves, force-displacement curves for the main local elements, pictures representing the collapse conditions.*
Data source location	*Italy, Europe.*
Data accessibility	*Data is with the article*
Related research article	Dall’Asta A, Leoni G, Morelli F, Salvatore W, Zona A, "An innovative seismic-resistant steel frame with reinforced concrete infill walls". Engineering Structures 141 (2017) pp. 144–158. http://dx.doi.org/10.1016/j.engstruct.2017.03.019.

**Value of the data**

• The force-displacement data presented within this paper can be used to compare the behavior of the specific dissipative SRCW system studied with similar earthquake-resistant systems.
• The data reports the experimental tests on two configurations of the SRCW system characterized by different distributions of the shear studs; data highlight their different influence on the global behavior of the prototypes.
• The detailed description of the test setup and of the behavior of the main elements allow to understand the behavior of the dissipative SRCW system leading to its further development.

## 1 Data

Prototypes of the SRCW systems were tested in the Official Laboratory of Material Experiences of the University of Pisa, Italy. The data provided within this paper summarize the experimental results achieved on an earthquake-resisting system: a dissipative Steel frame with Reinforced Concrete Wall (SRCW). The system is designed to dissipate the seismic energy through the plasticization of specific steel fuses placed within the steel columns; the infill wall is connected to the steel frame by connectors. The experimental data were used to validate the design method proposed in [1].

**Fig. 1:**
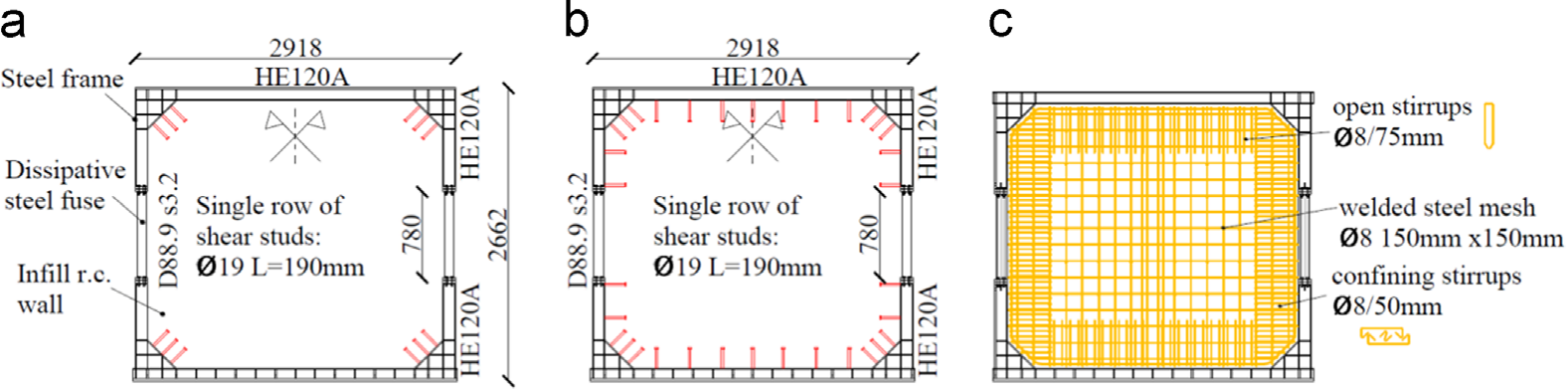
Overall geometry and shear studs’ distribution for the two SRCW specimens: a) *Configuration 1*, b) *Configuration 2* and c) reinforcement distribution.

The experimental tests were performed on two specimens characterized by different shear studs’ distributions. The first specimen, in the following referred as *"Configuration 1"*, represents the case in which the shear studs are used only to avoid the out-of-plane failure of the wall. The second specimen, referred as "*Configuration 2*", is characterized by a dense disposition of shear studs all over the steel frame, except for the zones close to the dissipative fuses, as presented in Fig. 1.

The reinforced concrete wall has, in both the two prototypes, thickness equal to 12 cm; the reinforcement layout, shown in Fig. 1**c**, is made up of a couple – one for each side of the wall – of welded steel meshes 150 mm × 150 mm of diameter 8 mm bars, supplemental confining reinforcements in the two vertical portions of the wall close to the dissipative elements and open stirrups all along the upper and lower edges of the steel frame.

## 2 Experimental design, materials, and methods

### 2.1 Experimental tests’ setup

**Fig. 2:**
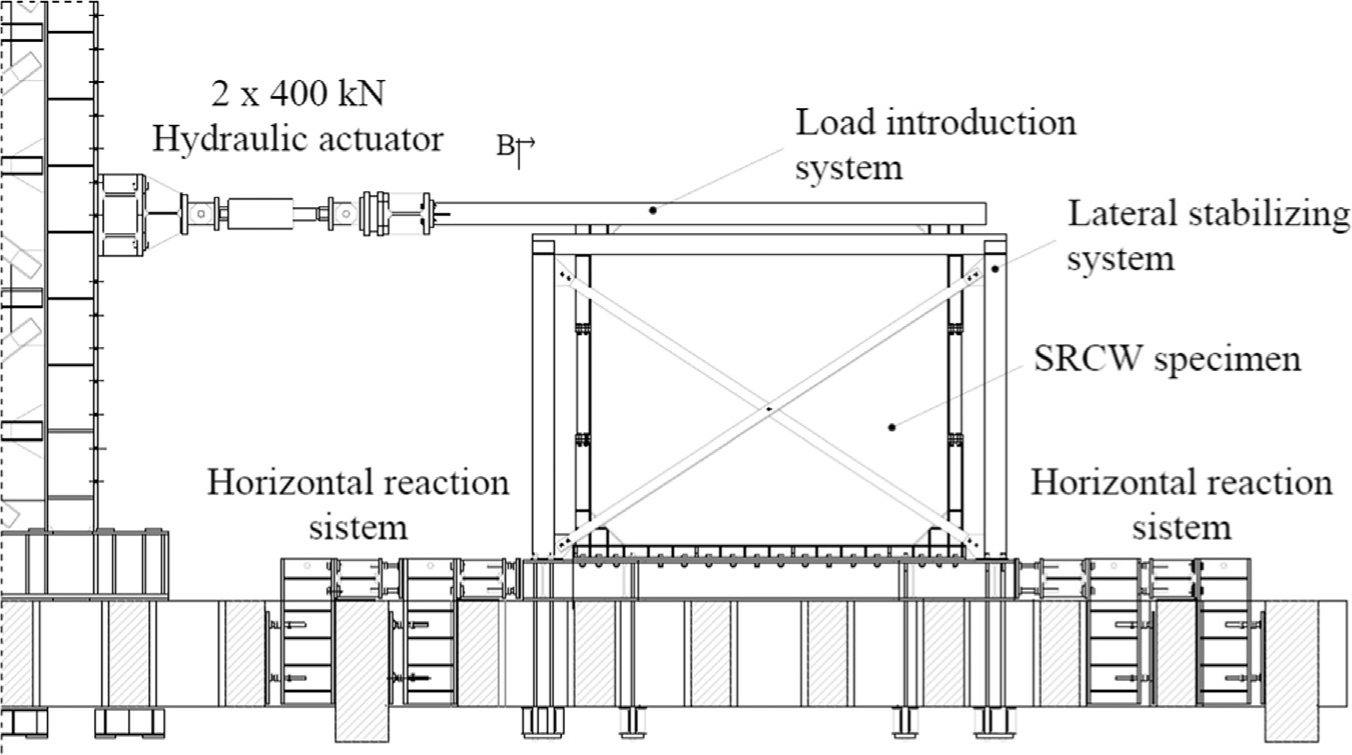
Overall experimental setup for the SRCW systems.

**Fig. 3:**
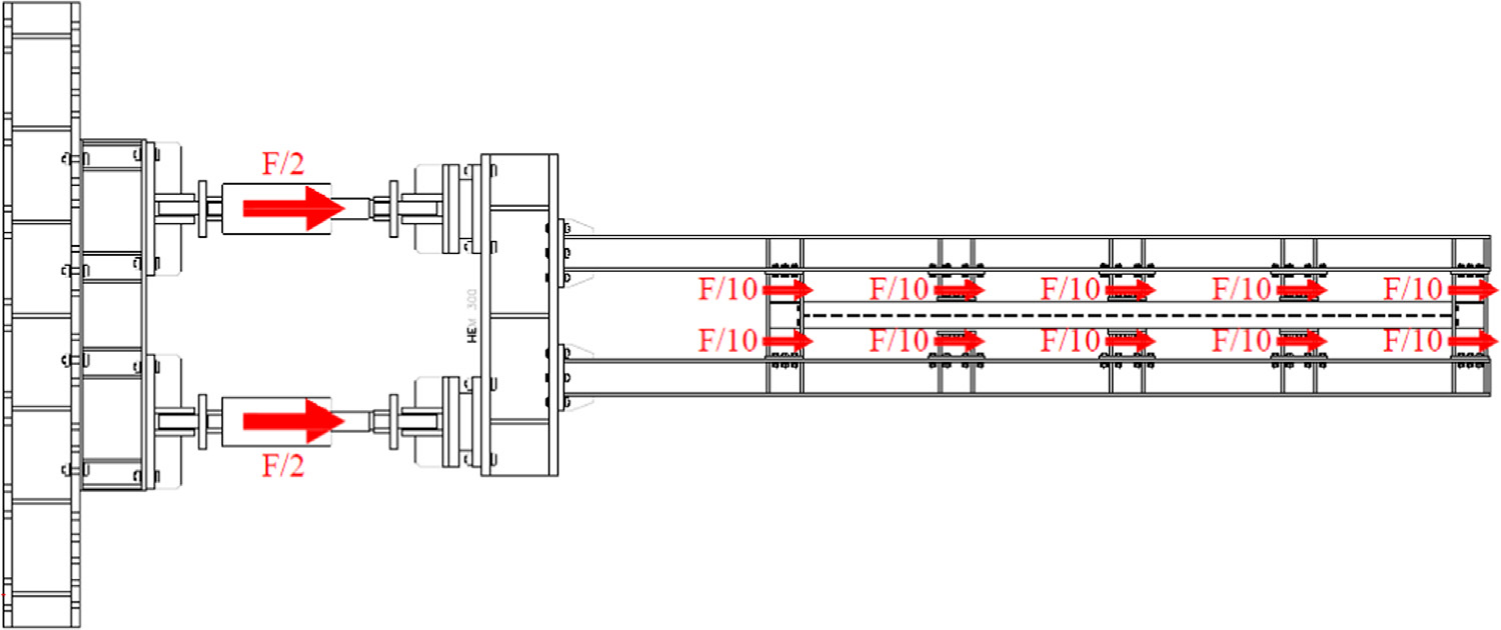
Loading distribution system.

The overall test setup is reported in Fig. 2. The SRCW specimen is bolted to a steel base firmly connected to the strong floor of the Laboratory of Pisa University by means of an anchor and a horizontal reaction system; a lateral stabilizing frame avoids the transversal displacements of the wall. To distribute the external force applied by the jacks all along the upper beam of the steel frame, the system presented by Fig. 3 is used. Such system connects the jacks to the specimen through 10 friction connections; the system is independent from the lateral stabilizing frame, then allowing the free tensile deformation of the dissipative elements.

**Fig. 4:**
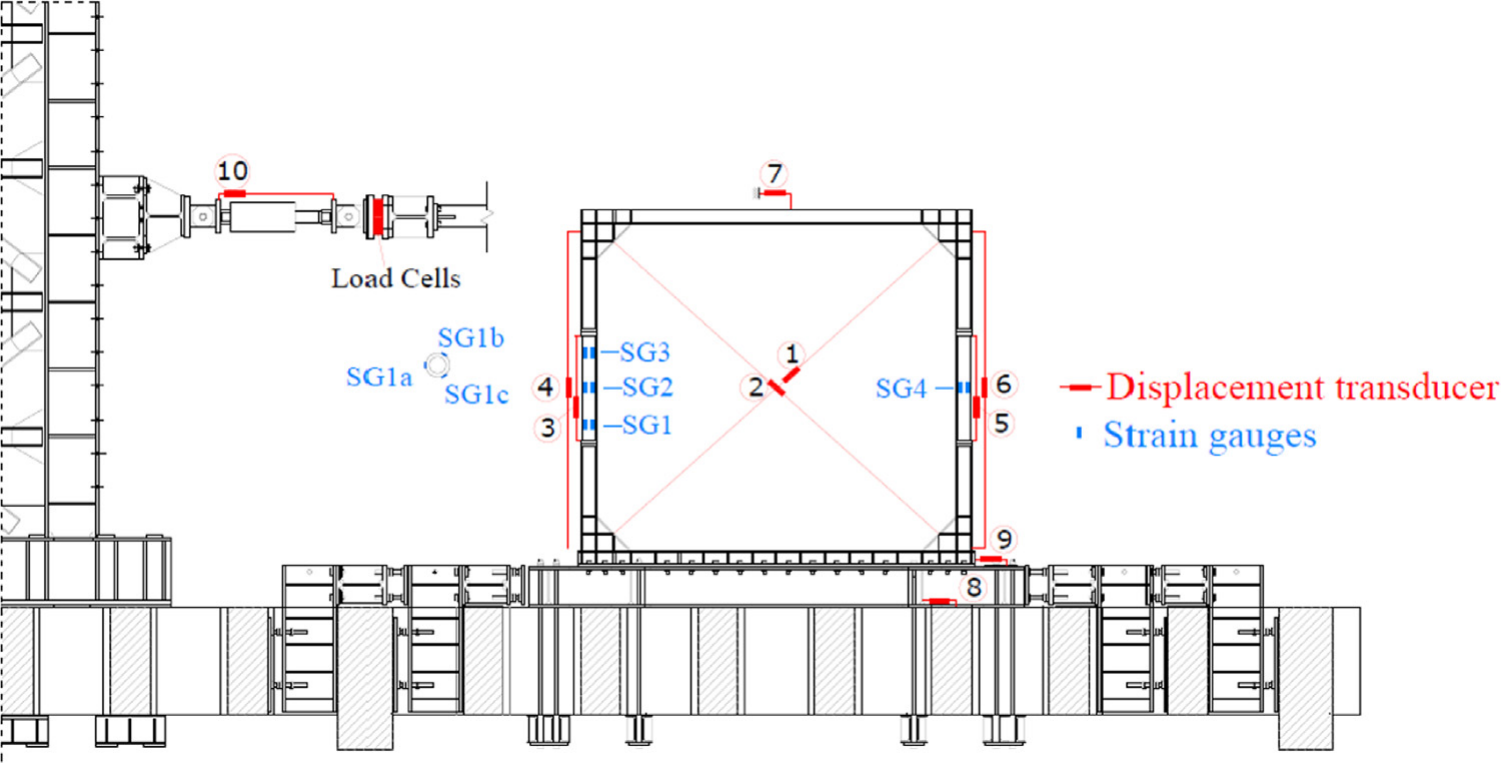
Load cells, displacement sensors and strain gauges distribution.

The displacements of the wall, the force applied, the deformation of dissipative elements and of the load introduction system are recorded by several sensors placed according to the disposition presented in Fig. 4. In particular:

• Displacement transducers #1 and #2 record the relative displacements between the opposite points of the wall diagonals.
• Displacement transducers #3 and #5 record the axial elongation of the dissipative links.
• Displacement transducers #4 and #6 record the vertical displacement of the vertical column constituting the steel frame.
• Displacement transducer #7 records the absolute horizontal displacement of the steel frame mid-span.
• Displacement transducers #8 and #9 record, respectively, the relative displacement between the steel base and the strong floor and the relative displacement between the SRCW frame and the steel base.
• Displacement transducer #10 records the horizontal displacement of the moving end of the jack.
• Nine additional strain gauges (three sets of three strain gauges – SG in the figure, with corresponding number) are placed on the dissipative fuse on the jack side and other three are located on the other one. Strain gauges record the axial deformation of the dissipative fuses.

**Fig. 5:**
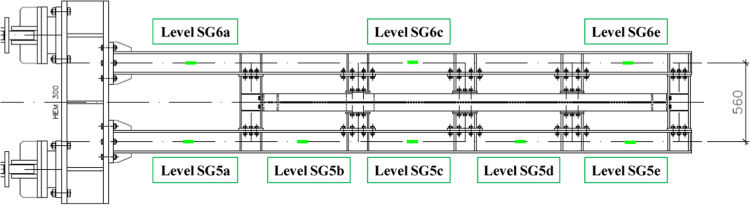
Strain gauges position along the load distribution system.

To estimate the real load distribution along the length of the steel frame beam, a series of linear strain gauges are applied also to the load distribution elements, as presented by Fig. 5. One side of the load distribution system is provided with five linear strain gauges that should allow the evaluation of the load amount transmitted by each connection element. The other side is provided with three linear strain gauges to assess the loading symmetry.

**Fig. 6:**
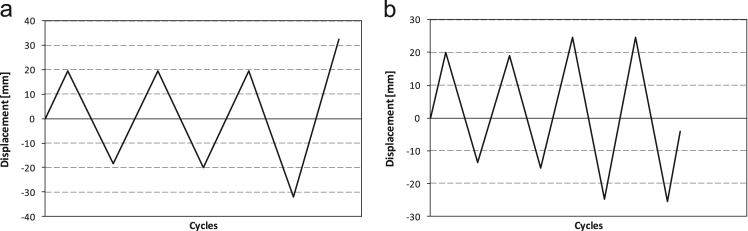
Imposed displacement vs time for Configurations a) 1 and b) 2.

Tests are carried out in displacement control and the displacement history imposed to the jacks end is reported, for both tests, in Fig. 6.

### 2.2 Experimental data

#### 2.2.1 SRCW Configuration 1

**Fig. 7:**
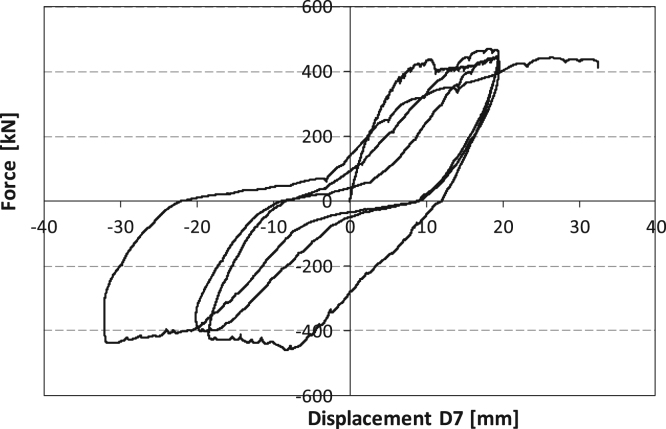
Force-displacement curve for the SRCW in configuration 1.

**Fig. 8:**
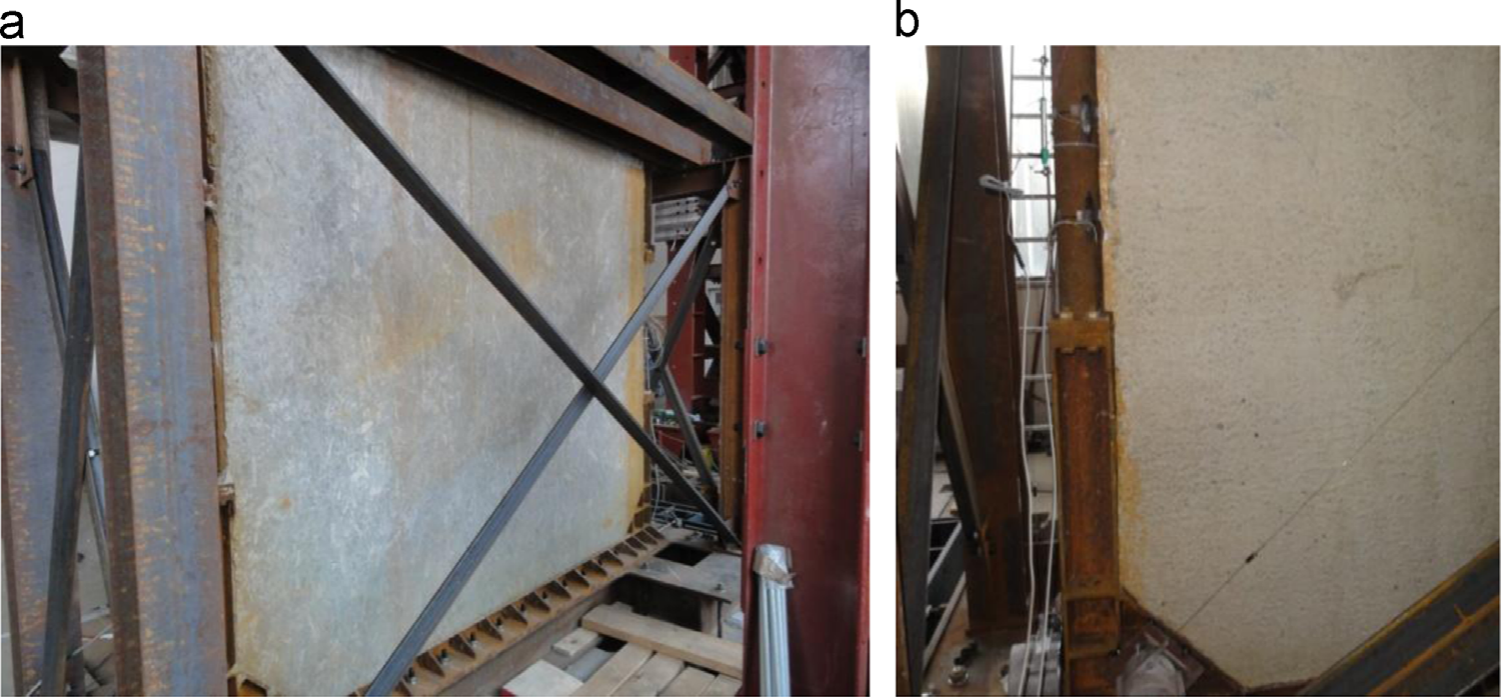
Condition of the specimen (Configuration 1) at the end of the first unloading phase.

Fig. 7 shows the experimental cyclic data recorded for SRCW (Configuration 1) by the load cells and the displacement sensor #7, according to Fig. 4. At the end of the first unloading phase, the concrete wall exhibits practically no damage, exception made for a little detachment from the lateral steel boundary elements, as presented by Fig. 8.

**Fig. 9:**
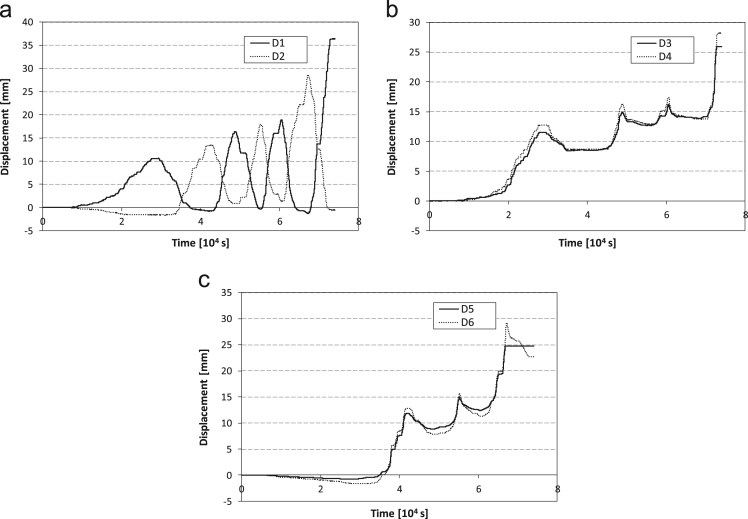
Displacement history for Configuration 1 recorded by a) the diagonal displacement sensors #1 and #2, b) the vertical sensors #3 and #4, c) the vertical sensors #5 and #6.

During the cyclic test, specimen 1 highlights the tendency to keep some plastic deformation in correspondence of the dissipative elements, vertical displacements (Fig. 9) are then cumulated in the lower interface between the steel frame and the infill wall. No cracks are detected within the concrete wall.

**Fig. 10:**
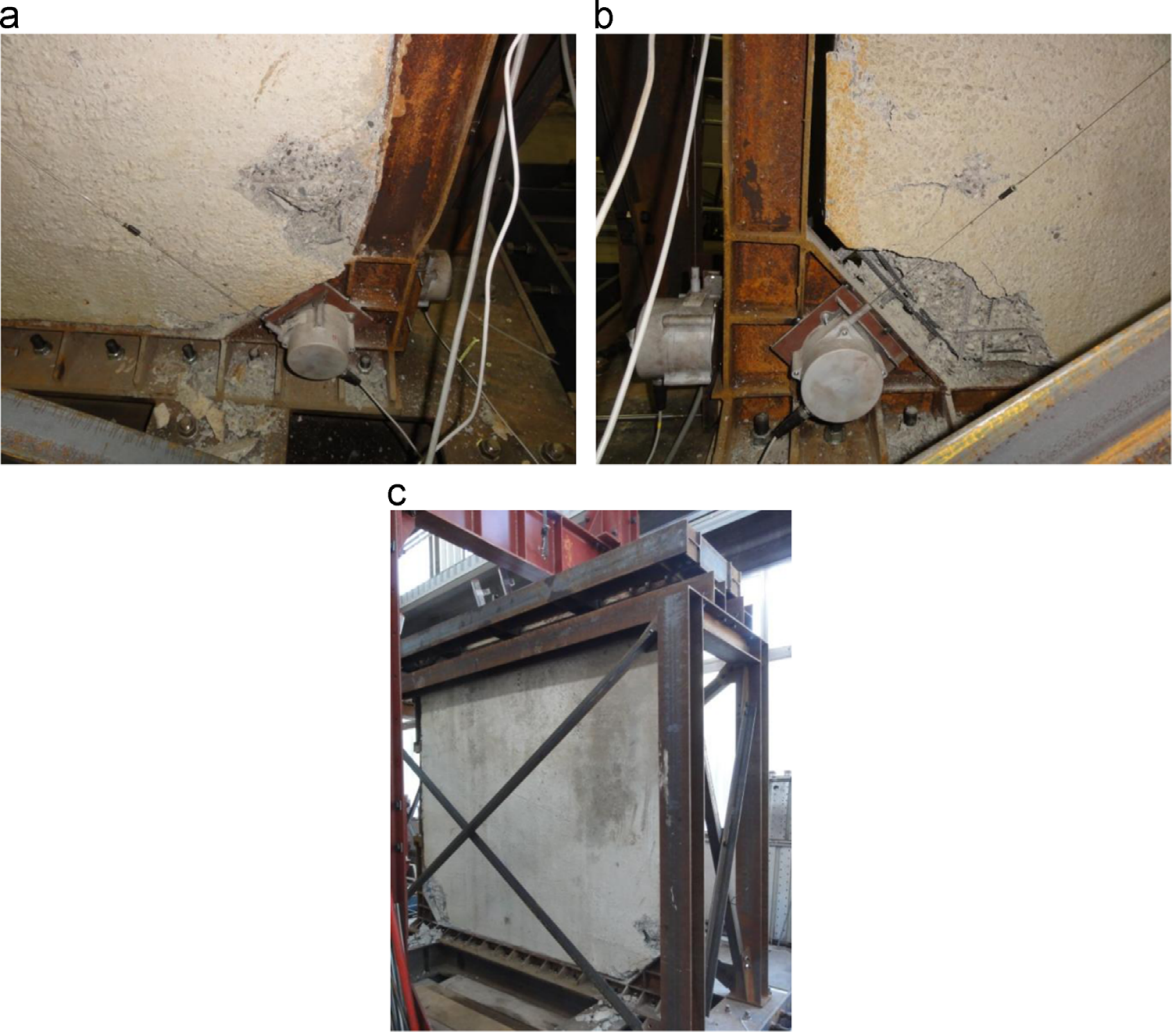
Lower corners of the Configuration 1 SRCW specimen after the failure: a) shear failure of the non-dissipative zone, b) spalling of the concrete and complete detachment by the steel frame and c) global view.

The failure of the specimen is due to an excessive shear deformation of the non-dissipative vertical steel element (Fig. 10**a**). At the same time, the spalling of the concrete on the opposite lower corner of the infill wall and the complete detachment of the infill wall from the steel frame occur (Fig. 10**b**). No other damages are visible within the reinforced concrete wall (Fig. 10**c**).

**Fig. 11:**
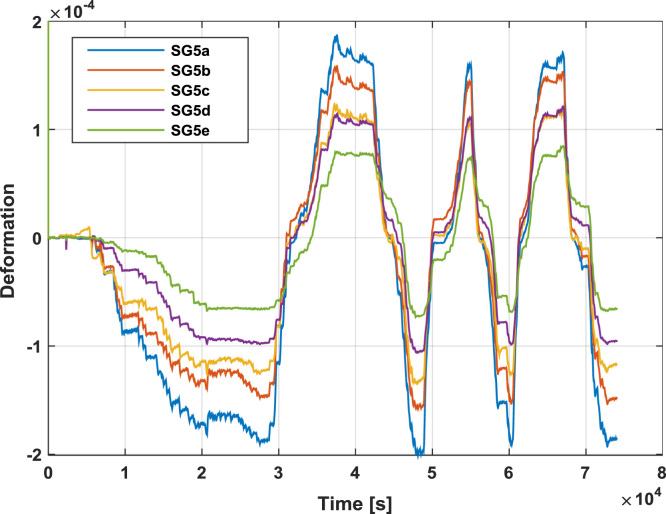
Strain histories recorded by the strain gauges placed on the load distribution system during the test on Configuration 1.

Fig. 11 reports the deformations recorded by the strain gauges placed on the load distribution system, according to Fig. 5.

#### 2.2.2 SRCW Configuration 2

**Fig. 12:**
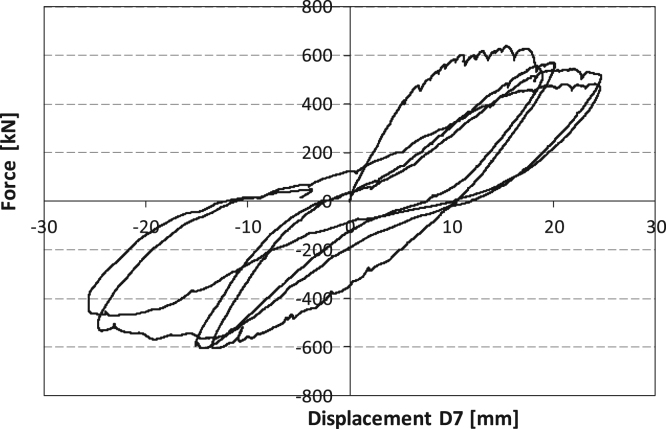
Force-displacement curve for configuration 2.

Fig. 12 shows the experimental cyclic data recorded for SRCW specimen (Configuration 2) by the load cells and the displacement sensor #7, see Fig. 4.

**Fig. 13:**
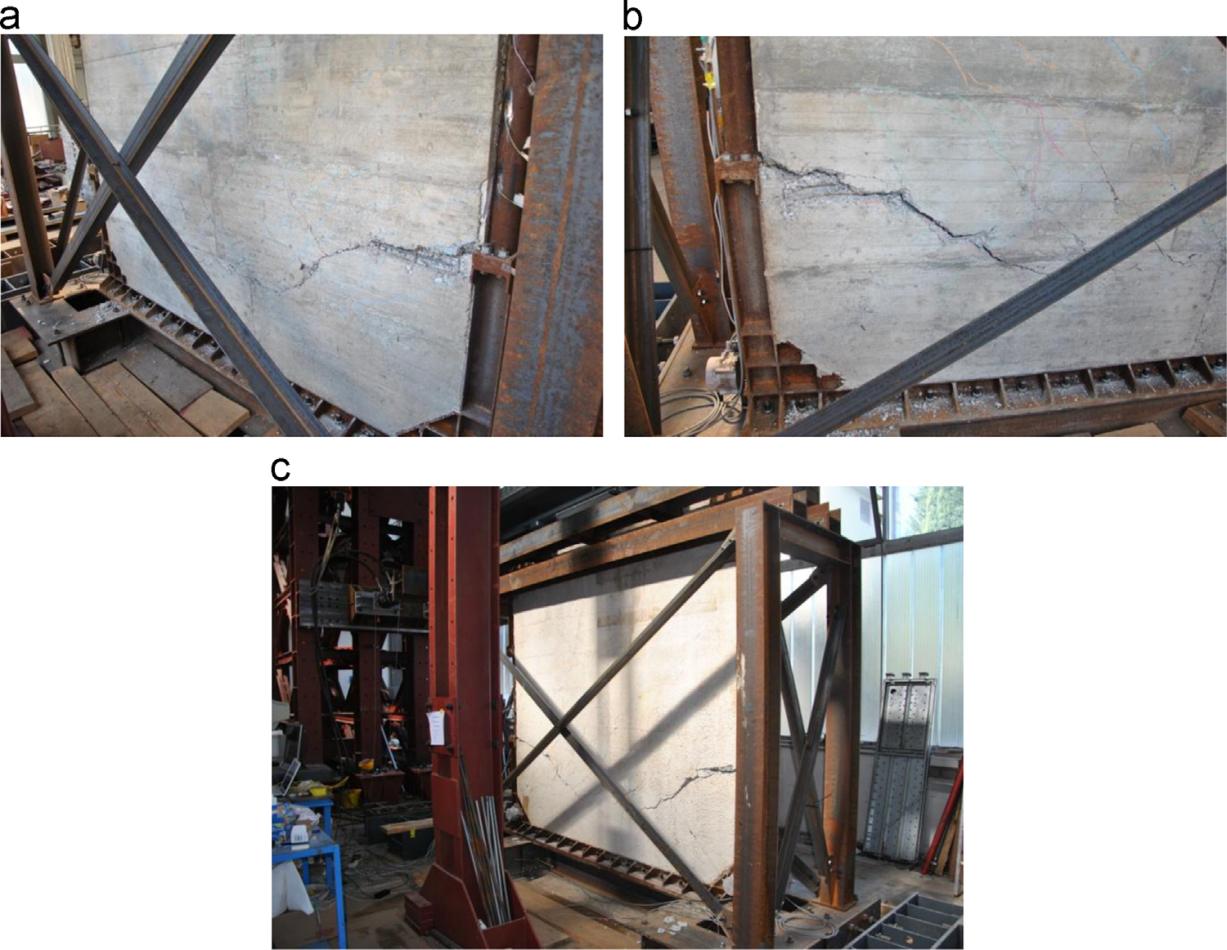
Cracking of the specimen.

The specimen is characterized by the propagation of main cracks from the base of the dissipative element in tension and by diffused cracking of the wall, as presented by Fig. 13; no detachment phenomena between the reinforced concrete wall and the steel frame are detected.

**Fig. 14:**
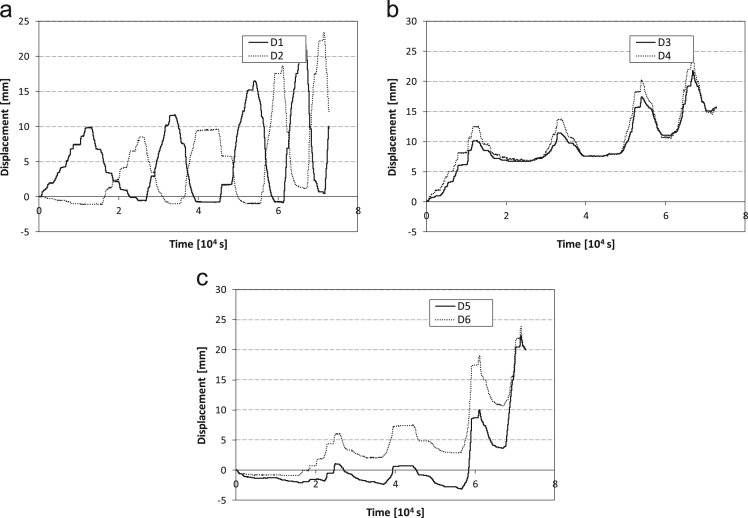
Displacement history for SRCW configuration 2 recorded by: a) the diagonal displacement sensors #1 and #2, b) the vertical sensors #3 and #4, c) the vertical sensors #5 and #6.

SRCW specimen in Configuration 2 shows, similarly to specimen 1, the tendency to accumulate some plastic deformations in correspondence of the dissipative elements (Fig. 14); the resulting vertical displacement causes, on the other hand, the gradual opening of the main cracks instead of the detachment of the wall from the lower edge of the steel frame.

**Fig. 15:**
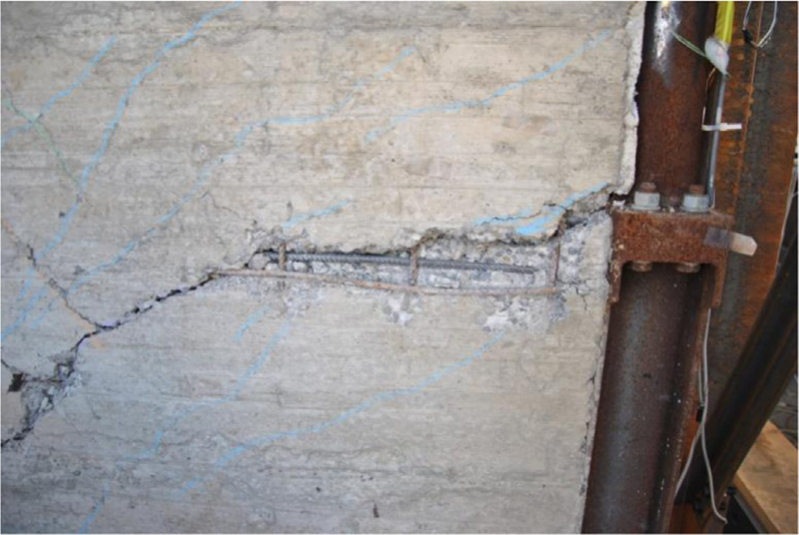
Failure of the reinforcing bars crossing the main crack.

**Fig. 16:**
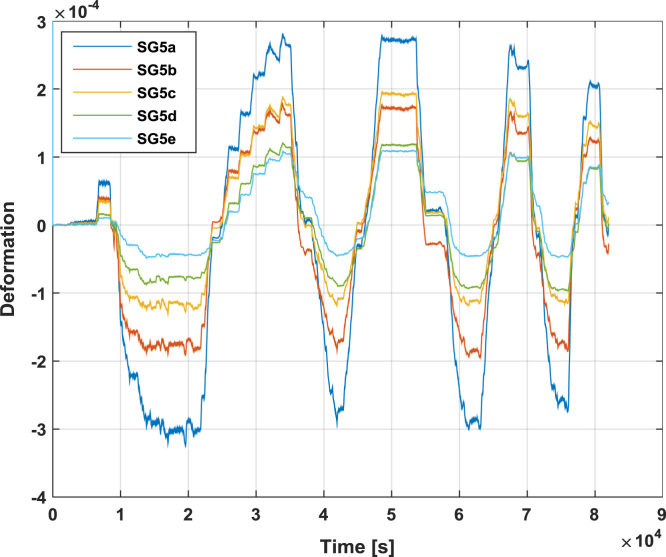
Strain histories recorded by the strain gauges placed on the load distribution system during the test on SRCW configuration 2.

The failure of the specimen is due to the failure of the vertical and horizontal reinforcing bars crossing the main crack (Fig. 15), causing the loss of some horizontal forces carrying capacity. Fig. 16 reports the deformations recorded by the strain gauges placed on the load distribution system (Fig. 5).
